# Performance of a lymphocyte t interferon gamma test (Quantiferon-TB gold in tube) in the diagnosis of active tuberculosis in HIV-infected children

**DOI:** 10.1371/journal.pone.0241789

**Published:** 2020-11-06

**Authors:** Bintou Sanogo, Alain Saga Ouermi, Makoura Barro, Anselme Millogo, Ad Bafa Ibrahim Ouattara, Ouédraogo Abdoul Salam, Boubacar Nacro

**Affiliations:** 1 Department of Pediatrics, University Hospital Center Souro Sanou (CHUSS), Bobo-Dioulasso, Burkina Faso; 2 Pediatrics Department, Regional Teaching Hospital of Ouahigouya, Ouahigouya, Burkina Faso; 3 Laboratory of Bacteriology-Virology, University Hospital Center Souro Sanou (CHUSS) Bobo-Dioulasso, Bobo Dioulasso, Burkina Faso; 4 Pediatrics Department, Regional Hospital of Gaoua, Gaoua, Burkina Faso; Food and Drug Administration, UNITED STATES

## Abstract

**Objective:**

Evaluate the performance of QuantiFERON ® -TB Gold In-Tube test (QFT-GIT), to improve the diagnosis of active tuberculosis (TB) in Human Immuno-Deficiency Virus (HIV)-infected children.

**Method:**

Sensitivity, specificity, Positive Predictive Value (PPV), Negative Predictive Value (NPV) of QFT-GIT were assessed in 58/63 HIV-infected children who were suspected of having TB.

**Results:**

Sensitivity of QFT-GIT was 20.69%, specificity 96.55%, PPV/NPV respectively 85.71% and 54.90%.

**Conclusion:**

QFT-GIT appears to be of little contribution to the diagnosis of active TB in children living with HIV in a TB-endemic country.

## Introduction

Tuberculosis is the leading opportunistic infection involved in the death of people living with HIV, justifying the importance of an early diagnosis in this group [1]. The diagnosis of TB is difficult in children, regardless of their HIV status, due to the difficulty of obtaining high-quality bacteriological samples [2–5]. The diagnosis is further problematic in case of co-infection with HIV due to the paucibacillary nature of TB infection in HIV/AIDS. Also, HIV infection may cause respiratory problems that can mimic tuberculosis clinically and/or radiologically. However, in the presence of respiratory symptoms it is crucial to consider tuberculosis in children infected with HIV; the incidence of tuberculosis being estimated twenty times higher than in uninfected children [2,4].

In the absence of microbiological evidence, in southern countries, the diagnosis is made based on factors such as the notion of exposure, the presence of suggestive symptoms, the demonstration of a specific immune response, and the radiological appearance [2]. Therefore, sensitive tools complementing conventional tests are needed to guide the initiation of therapy when the diagnosis of TB disease remains doubtful [6]. Interferon gamma release assays (IGRAs) are immunodiagnostics tools in which interferon-gamma (IFN-γ) released by T-cells in response to *Mycobacterium tuberculosis (M*. *tuberculosis)*-specific antigen is measured [4,6–8]. They are based on in vitro stimulation of T cells using antigens specific for *Mycobacterium tuberculosis*, which are absent in Bacillus Calmette and Guérin (BCG) and in most atypical mycobacteria. These proteins are "early secreted antigenic target 6 [ESAT-6]", "culture filtrate protein 10 [CFP-10]" ± TB7.7. Two tests are commercially available: the QuantiFERON-TB® Gold In-Tube test (QFT-GIT, Cellestis Limited, Carnegie, Victoria, Australia) and the T-SPOT TB (T-SPOT®, Oxford Immunotec Ltd. UK**).**

The QFT-GIT has been reported to have higher sensitivity, specificity and be more attractive than the tuberculin skin test (TST) [4–7,9–11] which may result in interpretation errors [5,6,11], including the need of two clinic visits. Collecting blood samples may not frequently encounter difficulties compared with other specimens collection and QFT-GIT can provide rapid results, within two days [6]. However, specificity depends on multiple factors in addition to the test antigen, including the cutoffs used to interpret the test and the analytical methods employed to measure IFN-γ concentrations [7]. Also, it’s performance in detecting active or latent tuberculosis infection (LTBI) can vary depending on the tested populations and regions [6]. Numerous studies have assessed the utility of the QFT-GIT in diagnosing latent TB infection (LTBI) in various clinical settings [6]. Regarding active TB, the use of IGRAs has been unclear particularly in HIV-infected children in high prevalence settings [4,6,12–16]. This work aims to evaluate the contribution of QFT-GIT in the diagnosis of active TB in children infected with HIV and TB suspects in Bobo Dioulasso, a TB endemic country (49 per 100000 data from 2018) [17].

## Methodology

We analyzed data of University Hospital Center Souro Sanou (CHUSS) of Bobo-Dioulasso, participating in a multicentric prospective cohort study ANRS 12229 (Agence Nationale de Recherche sur le Sida): PAANTHER 01 (Pediatric Asian African Network for Tuberculosis and HIV Research). This multicentric study was simultaneously conducted at 7 other hospitals sites in Cambodia, Cameroon and Vietnam [3]. At Bobo Dioulasso (Burkina Faso), the study took place from December 2012 to November 2014.

### Study design and participants

#### HIV-infected children

After an informed consent was signed by parents or guardians, were included in the study, 63 HIV infected children aged 0 to 13 years, on anti-retroviral (ARV) treatment or not, with a suspicion of intrathoracic tuberculosis based on at least one of the following: cough and/or fever over 2 weeks, failure of broad spectrum antibiotics for a pulmonary infection; or suggestive chest radiograph anomaly. Those with a history of TB treatment started less than 2 years before inclusion were excluded. For each child we had collected: socio-demographic data (age in years, gender), anthropometric data (weight, height, weight-for-age Z score < −2 standard deviation reflecting failure to thrive; the Z score expresses the deviation from the mean value), medical history (BCG vaccination, TB exposure or previous TB), suspected clinical signs of TB, ARV treatment.

#### Procedures [3]

**QFT-GIT:** A sample of 1 ml of whole blood per tube (antigen, zero, mitogen, tubes delivered by Cellestis QIAGEN Company) was collected for QFT-GIT. Immediately after collection, each tube was shaken ten (10) times from top to bottom and then sent to the laboratory, where they were incubated at 37° C for 24 hours. Then the plasma (about 400 microliters) was taken after centrifugation at 3,000 rpm for 15 minutes and stored at -20° C until assaying QFT-GIT by the ELISA technique. The optical density was measured using a Thermo Labsystems Multiskan Ex brand microplate reader. The results were classified as positive, negative or undetermined according to the manufacturer's recommendations [18]. Due to tubes expiration, QFT-GIT was performed in 58 of the 63 patients.

**Tuberculin-skin test (TST):** For TST, intradermal injection of 5IU of Purified Derivative Protein (Tubertest Aventis, Pasteur MSD, Lyon, France) was performed on the forearm. Induration ≥ 5 mm after 72 hours was considered positive.

**CD4 count:** CD4 counts in whole blood were measured by flow cytometry at entry and at 6 months thereafter using the Facs Counttest (Beckman Coulter, inc. Marseille, France) according to the manufacturer’s instructions.

#### Viral load (VL)

Plasma viral loads were measured at a participant’s first presentation to our center and at 6 months thereafter using the COBAS® Ampliprep/COBAS® Taqman® HIV-1 Ts7 v2.0 (Roche Diagnostics, Maylan, France) using m2000sp/rt (Abbott Molecular Inc., 1300 East Touhy Avenue, Des Plaines, IL 60018, United States of America) according to the manufacturer’s instructions. The lower quantification limit was 300 copies/mL.

#### Other tests

We also performed for each child: chest x-ray, and an abdominal ultrasound to explore for signs of TB.

#### Bacteriological samples

Based on the age, we performed:

- collection of standard bacteriological samples (3 gastric aspirates for children under 4 years of age on 3 consecutive days, 2 gastric aspirates for those 4–10 years old over 2 days, and 3 expectorated sputum samples for those over 10 years old for 2 days)
- collection of alternative bacteriological samples: for children over 4 years old, a string test (a nylon thread with a gelatin capsule at the end) was performed on day 3, using the Pediatric Entero-test® (HDC Corporation, Milpitas, California, USA), an FDA-approved device for collection of gastric contents in children for detection of parasites and bacteria (Helicobacter Pylori) [3].

Stool and nasopharyngeal aspiration are other alternative bacteriological samples performed for all children.

**Tuberculosis diagnostic techniques:** All bacteriological samples were decontaminated with N-acetyl-L-cysteine and sodium hydroxide and centrifuged; stool samples were previously prepared by dilution in sterile water. Centrifuged pellets were resuspended in phosphate buffer.

**Microscopy:** Direct exam was performed using the Ziehl–Neelsen method for stool samples and fluorescent (auramine) microscopy from a drop of pellet suspension was performed for all other samples.

**Culture:** For culture, sample pellet suspensions were inoculated in Lowenstein–Jensen slants. Isolates were identified as Mycobacterium tuberculosis complex by Ziehl–Neelsen staining and biochemical method.

**GeneXpert® MTB/RIF (Xpert® MTB/RIF):** The Xpert® MTB/RIF assay is a single cartridge-based test for rapid and simultaneous diagnosis of tuberculosis and multi drug-resitant TB (MDR-TB). It was performed on 1.0 mL of fresh sample or decontaminated pellet if the sample volume was not sufficient, following the manufacturer’s recommendations [19]. Results were automatically generated within 2 hours and reported as MTB-detected or non-detected and RIF susceptible or resistant. The former determination is based on the amplification of any two rpo*B* gene regions, and the latter determination is based on a difference of >3.5 amplifications cycles of any probe. Stool samples were previously processed by emulsification of 0.5 g of material in Sheather’s solution (28% sucrose in distilled water), filtered through funnel gauze, and centrifuged [3].

**TB disease diagnosis:** TB disease diagnosis was assigned to any child with *M*. *tuberculosis* positive culture or detected by microscopy or GeneXpert**®** MTB/RIF assay from any bacteriological sample. For children it is often difficult to obtain a positive result from the above investigations. As a consequence, the TB disease diagnosis was also assigned to any child with clinical and radiologic evidence of active TB, and with either a history of exposure to an infectious.

Each child was followed for 6 months. Antiretrovirals and antituberculosis treatment were initiated per national guidelines and provided by national programs.

**Statistical analyzes:** The data were entered and analyzed on a laptop using the Stata 11 software. The Chi square and Fisher exact (if n <5) tests were used with a significance level of 5%. We used frequency for the qualitative variables, the median and the inter quartile interval (IQR) for the quantitative variables. QFT-GIT assay sensitivity was defined as the proportion of positive results identified among tuberculosis case (based on anamnestic, clinical, bacteriological and radiological data). Indeterminate results were included in calculating sensitivity estimates.

#### Clinical Trials Registration

The study was approved by Burkina Faso ethics committee for health research (number 2011-8-47 of 03 August 2011). The ANRS 12229 PAANTHER 01 Study is registered at ClinicalTrials.gov (identifier NCT01331811).

## Results

### Baselines characteristics

Sixty-three HIV-infected children suspected of having TB were included in the study. Baselines characteristics are shown in Table 1. More than half of patients had BCG scar (71.4%) and 12.7% had history of TB more than 2 years. Thirty-seven (58.7%) children were in stage III HIV infection based on the revised 2014 (Centers for Disease Control and Prevention CDC) recommendations of VIH classification [20], and 52.38% were on antiretroviral treatment at inclusion. All 32 children who were TB negative had no sign of active tuberculosis during the 6-months follow-up.

**Table 1:** Baseline characteristics.

Variables	Sub-group	Value (n = 63)
Age (year): median (IQR)		8 (6–10)
< 4 years		7 (11.11%)
4–9 years		33 (52.38%)
10–13 years		23 (36.51%)
Sex	Female	33 (52.38%)
BCG Vaccination	Received	51 (80.95%)
	Not Received	3 (4.76%)
	Undocumented	9 (14.29%)
BCG Scar	Present	45 (71.43%)
	Absent	18 (28.57%)
History of TB more than 2 years	Yes	8 (12.7%)
	No	55 (87.30%)
Type of previous TB		1 (12.5%)
		3 (37.5%)
		2 (25%)
		2 (25%)
TB exposure		10 (15.87%)
		10 (100%)
		0
People exposed to TB at home		6 (60%)
		4 (40%) ^a^
Malnutrition (WA Z score< –2)		26 (41.27%)
WHO HIV clinical staging:		
Stage I		5 (7.9%)
Stage II		15 (23.8%)
Stage III		37 (58.73%)
Stage IV		6 (9.52%)
Patients on ARV treatment at inclusion		33 (52.38%)
Frequency of TB confirmed to bacteriology children confirmed TB to bacteriology:		31 (49.2%) 13 (41.93%)
	PTB+	
	PTB-	
	Extra pulmonary TB	
	Undocumented	
	Total	
	At home	
	Away from home	
	Mother	
	Other person	

BCG: Bacille Calmette Guérin; TB: Tuberculosis; PTB+: Microscopic positive pulmonary tuberculosis; PTB-: Microscopic negative pulmonary tuberculosis, WA Z score: Weigh for age Z score; WHO: World Health Organization; HIV: Human Immuno-Deficiency Virus, ARV: Anti-retroviral, IQR: Inter quartile interval.

### Bacteriology results

Basis of anamnestic, clinical and paraclinical (bacteriological, radigraphic, ultrasound) arguments, the frequency of active TB was 49.2% (31/63). In the 31 patients with active TB, we collected 167 standard and alternative bacteriological samples. Twenty one (21) samples were positive at bacteriology: 2 samples (2 gastric aspiration of the same child) at microscopy, 7 samples (3 gastric aspiration, 2 stools, 2 nasopharyngeal aspiration) at culture, and 12 samples (3 gastric aspiration, 4 nasopharyngeal aspiration, 2 septum, 2 string test, 1 stool) at Xpert® MTB/RIF. These 21 positive samples at bacteriology were results of 13 patients (each patient had have at least 5 samples; see methodology). One patient was positive on both microscopy and Xpert® MTB/RIF. And two positive patients for both culture and Xpert® MTB/RIF. The results of bacteriology are summarized in the Table 2.

**Table 2:** Microscopy, cutlure and GeneXpert® MTB/RIF results in patients with active TB.

Results	Microscopy	Culture	Xpert^®^ MTB/RIF
Positive	1 **(3.23%)**	6 **(19. 35%)**	8 **(25. 81%)**
Négative	30 (96.77%)	25 (80.65%)	23 (74.19%)
Total	31 (100%)	31 (100%)	31 (100%)

One patient had positive samples both at microscopy and at Xpert® MTB/RIF. Two patients had positive samples both at culture and Xpert® MTB/RIF.

### QFT-GIT results

QFT-GIT was performed in 58 of the 63 patients, and the results were as follows: 12% (7) positive, 52% (30) negative, 36% (21) undetermined. Of the thirty-one (31) patients classified as having active tuberculosis, QFT-GIT was performed in twenty nine (29) cases. Among them, six (6) patients had a positive QFT-GIT result, giving a QFT-GIT confirmation rate of 20.69%. One (1) positive QFT-GIT patient was not classified as having active TB. Table 3 shows the results of the 7 Positive QFT-GIT according of microscopy, culture and Xpert® MTB/RIF.

**Table 3:** Results of the 7 Positive QFT-GIT according of microscopy, culture and Xpert® MTB/RIF.

	Microscopy	Culture	Xpert® MTB/RIF
Positive QFT-GIT			
Patient number 1	Negative	**Positive**	Negative
Patient number 2	Negative	Negative	**Positive**
Patient number 3	Negative	Negative	Negative
Patient number 4	Negative	Negative	Negative
Patient number 5	Negative	Negative	Negative
Patient number 6	Negative	Negative	Negative
Patient number 7	Negative	Negative	Negative

QFT-GIT: QuantiFERON® -TB gold in-Tube test, Xpert® MTB/RIF: Gene Xpert® MTB/RIF.

The distribution of the six (6) positive QFT-GIT/active TB cases according to the elements of our gold standard (medical history, clinical, imaging, bacteriology), was:

- two (2) were confirmed by bacteriology testing (1 in stool culture; 1 at Xpert® MTB/RIF of nasopharyngeal aspiration).
- One (1) had images of patent miliary TB on chest x-ray.
- Three (3) patients were classified as having active TB based on medical history and clinical criteria.

There was no significant association between the QFT-GIT results (positive/negative) and certain factors: age less than 2 years (Fisher exact = 0.705), sex (Fisher exact = 0.687), ARV treatment (p = 0.20), CD4 count (p = 0.18), and viral load (p = 0.67). On the other hand, there was a significant difference between QFT-GIT results (positive/negative) and other factors: BCG vaccination (p < 0.001), nutritional status (Z-score weigh for age < -2SD) (p = 0.04), HIV stage III (p = 0.04). Table 4 described QFT-GIT results according to factors such as BCG and nutritional status, HIV stage, CD4 count and viral load. The median CD4 counts were 740 cells/μL (IQR: 227–872).

**Table 4:** QFT-GIT results according to BCG status, nutritional status, CD4 count and viral load.

	All (n = 58)	QFT-GIT det (n = 37)	QFT-GIT ind (n = 21)	P value	QFT-GIT pos (n = 7)	QFT-GTI neg (n = 30)	P value
**BCG status:**							
Documented, n (%)	50 (86%)	32 (87%)	18 (86%)	0.93	6 (86%)	26 (87%)	0.94
Vaccinated among documented, n (%)	47 (94%)	32 (100%)	15 (83%)	**0.01**	6 (100%)	26 (100%)	**< 0.001**
**Status at QFT-GIT sampling:**							
Weigh for age below 2 SD, n (%)	23 (40%)	12 (32%)	11 (52%)	0.13	-	12(40%)	**0.04**
**HIV stage**							
Stage III among documented, n (%)	32 (55%)	19 (51%)	13 (62%)	0.43	6 (86%)	13 (43%)	**0.04**
**CD4 count: cells/l, median (IQR**)	740 (227–872)	748 (441–859)	314 (48–798)	0.16	754 (745–809)	734 (441–859)	0.18
**Viral load: copies/ml, median (IQR)**	35296 (0–671511)	2404 (0–396543)	57971 (1165–899740)	0.17	0 (0–4440000)	11252 (0–396543)	0.67

BCG: Bacille Calmette Guérin; SD: Standard deviation; QFT-GIT det: QuantiFERON® -TB gold in-Tube test determined; QFT-GIT ind: QuantiFERON® -TB gold in-Tube test indeterminate; QFT-GIT pos: QuantiFERON® -TB gold in-Tube test positive; QFT-GIT neg: QuantiFERON® -TB gold in-Tube test negative; IQR: Inter quartile interval.

Concerning the performance of the QFT-GIT, its sensitivity was 20.69%, its specificity was 96.55%, the PPV/NPV were 85.71%/54.9%, respectively.

Table 5 summarizes the different analytical performances of the four (4) diagnostic techniques used.

**Table 5:** Summary of the different analytical performances of the four (4) diagnostic techniques of TB.

Test		Performance (95% CI)		
	Se	Sp	PPV	NPV
**Microscopy**	33% [-2% - 9%]	100% [100% - 100%]	100% [100% - 100%]	51.61% [37% - 67%]
**Culture**	19.35% [8% - 31%]	100% [100% - 100%]	100% [100% - 100%]	56.14% [41% - 71%]
**Xpert® MTB/RIF**	25.81% [15% - 37%]	100% [100% - 100%]	100% [100% - 100%]	58.18% [46% - 71%]
**QFT-GIT**	25% [11% - 39%]	83% [71% - 95%]	29% [14% - 44%]	80% [67% - 93%]

CI: Confidence interval; Se: Sensitivity; Sp: Specificity; PPV: Positive Predictive Value; NPV: Negative Predictive Value; QFT-GIT: QuantiFERON® -TB gold in-Tube test, Xpert® MTB/RIF: Gene Xpert® MTB/RIF.

### TST results

The TST performed for 52 patients was negative in 100% of cases. All 31 patients with active TB had negative TST.

## Discussion

This study evaluated the performance of QFT-GIT assay in improving the diagnosis of active TB in HIV infected children. Our data showed higher incidence of active TB (49.2%) compared to others [4,21], probably because of the HIV status of all our patients and the new diagnostic methods used in our study. Indeed, the incidence of TB is 20 times higher in children infected with HIV [2]. More sensitive diagnostic techniques, including Xpert® MTB/RIF, auramine-stained fluorescence microscopy, and new methods for collecting bacteriological samples (nasopharyngeal aspiration, string test, stool) should help to better account for the prevalence of tuberculosis in children infected with HIV/AIDS.

Current pediatric guidelines recommend applying a combination of clinical, radiological, microbiological and immunological approaches to improve the diagnostic yield of TB [22–24].

Regarding IGRAs, although some studies have shown that they are more precise than TST in children [25–27], their systematic use is not yet approved, especially in children with an immune deficiency, such as children under 5 years of age and infected HIV children [28–30].

In our study, we found a high negative (52%) and undetermined (36%) QFT-GIT results. The same observation is made by other authors: QFT-GIT negative 96% [1], 80.25% [31]_,_ and 80.9% [32]; QFT-GIT undetermined (35% and 27% respectively) [8,12]. These high levels could be explained by the immunosuppression of patients in our work. Other authors found indeterminate and negative QFT results lower than our results [4,5,21,26,30]. We have evaluated performance of QFT-GIT in all children diagnosed as having active TB, regardless of bacteriological confirmation, and the main result of this work is the low sensitivity of QFT-GIT results (20.69%).

Our results are consistent with those of Michala in Tanzania (19%) **[33]**. Hormi in her study in France has shown that QFT-GIT has a lower sensitivity in HIV-infected children compared to HIV-uninfected children (29% versus 94%, respectively; p = 0.002) **[4]**. Bamford in his study on 333 children with 195 active TB (49 confirmed at bacteriology) and whose HIV status was not specified, has shown that QFT-GIT may have limited sensitivity in diagnosing active TB [8]. Possible explanations of the low sensitivity of QFT-GIT in children are, on one hand, the low production of cytokines such as interferon gamma in children, even after infancy [33]; on the other hand, the antigenic response specific to *M*. *tuberculosis* infection is delayed and less effective in children compared to adults [34]. It is likely that low levels of TB antigen-specific IFNγ secreting T cells in addition to more generalised T cell immune suppression observed in active disease might lead to impaired responses in IGRA, especially in children [8].

Compared to Winsley’s study in Toronto [31], we have found no significant association between the QFT-GIT results and some of the factors described in the literature, such as young age, gender, ARV treatment. Tao Li [5] in China have not found significantly difference in the results of QFT-GIT between children less than 5 years old and those more than 5 years old [5]. Chiappini [30] in her review concerning non infected HIV children in Italy, has found the high sensitivity of QFT-GIT (85.3%), that tended to be lower in young children, 81.8% (95% CI: 65.7–97.9) in < 2 years old and 82.4% (95% CI: 72.6–92.3) in 2–4 years, compared to those aged 5–18 years (87.6%) (95% CI: 81.3–93.9). She found that the sensitivity of QFT-GIT was very good in children with microbiologically confirmed active TB, not only in those older than 5 years (89.1%) but also in children aged 2–4 years (95.0%). But she has not considered some confounding factors at multivariate analysis such as malnutrition [30]. In her previous study on 338 children [29] (with 28 cases of microbiologically confirmed TB), the sensitivity of QFT-GIT in children younger than 5 years of age was 73.3%. The performance of QFT-GIT in the study of Debord et al. [35] was 79% in the same age group. The conflicting results have been reported in litterature concerning sensitivity of QFT-GIT and young age [5,26,36,37]. A study conducted by Kay and all at US [36], QFT-GIT sensitivity was 91% in children aged 2–4 years but only 80% in those < 2 years old. Conversely Petrucci et al [26], reported a 93% QFT-GIT sensitivity in children less than 2 years of age. In Velasco- Arnaiz’s cohort study of children aged < 5 years the sensitivity QFT-GIT in 39 children with active TB (16 microbiologically confirmed) was 93.7%. However, the sensitivity in those aged less than 2 years was not reported [37]. Tao-Li at Shanghai has found the high sensitivity (83.9%) of QFT-GIT for the diagnosis of childhood TB, but not inflenced by age [5].

Beside age, HIV infection [1,12,38–41], and malnutrition are likely to interfere with the sensitivity of QFT-GIT, due to immunosuppression and suppression of the response of T cells [16–19]. Thus a high viral load, as well as low CD4 levels were associated with a decrease in sensitivity of the QFT-GIT in children [1,38]. We have found a significant association between nutritional status (Weigh for age below—2 SD) (p = 0.04), HIV stage III (p = 0.04), BCG status (p<0.001) and QFT-GIT positive and negative results; but not association between CD4 count, VL and QFT-GIT results. Hormi in her limited series of co-infected children, an adverse effect on QFT-GIT positivity of low CD4 cell counts was not obvious [4]. The most likely explanation for false negative results in children with normal or moderately decreased CD4 counts is the impaired functional capacity of these cells, especially for children at stage III of HIV infection [4]. Otherwise, in low-income countries, children are brought in consultation after long periods of illness causing a weakened immune system. These data suggest that the performance of QFT-GIT in these children is affected by the immaturity or failure of the immune system, due to the progressive loss of T cells’ ability to respond adequately to antigens [42]. None of the children with a history of TB had a positive QFT-GIT. This could be explained on one hand by a probable reversion of IGRAs (transition from positivity to negativity) following treatment with anti-TB drugs [1,38]; indeed, IGRA reversion is noted after 6 months of treatment with rifampicin and isoniazid [1]. On the other hand, it is possible that these children were wrongly diagnosed with TB; the diagnosis of TB is difficult in children in general, especially in those infected with HIV [1]. We found a specificity of QFT-GIT of 96.55%. High specificity of QFT-GIT is also found in other studies: 88.5% [5], 85% to 97% [10], 90% [33], 94% [25], 99.3% [26]. IGRAs tests need high specificity to minimize unnecessary treatment and high sensitivity to allow maximum detection and prevention of TB [7].

Our results suggest that a negative IGRA should not be used to rule out TB infection.

The PPV of QFT-GIT in our work (85.71%) is well above the 6.8% found in the high-risk subgroup of TB in the Diel meta-analysis [43], but lower than 92.9% of Li [5]. However, our NPV is lower than that found by the same authors (82.1% and 99.7% respectively) [5,43]. These differences could be explained by the high prevalence of TB in Burkina Faso and because of the immunocompromised status of HIV patients in our study.

The TST was negative in 100% for children who had it performed, while 71.43% of the children had a BCG scar, and 15.87% a notion of TB exposure. Our results could probably be explained not only by technical errors during the test, but also by the immunocompromised status of the patients. TST is a reliable diagnostic tool if the technique is well applied, and the conditions of interpretation are respected. The disagreement between TST and IGRA results could be a consequence of the distinct immunologic mechanisms responsible for positive TST or IGRA tests. TST is a delayed-type hypersensitivity reaction that measures both effector and memory T cells function, whereas IFN- gamma, with its short period of incubation, measures mostly the effector T-cell function. Thus, a positive IGRA result may indicate a more recent or ongoing TB infection, whereas a positive TST result could indicate a more remote TB infection [21].

The small size of our sample did not yield highly significant results for most factors associated with QFT-GIT results in the literature. The concomitant use of the TST has not been conclusive, in view of the negativity of all tests. Other limitation is the absence of microbiologic confirmation in a substantial proportion of active TB cases. *M*. *tuberculosis* detection is the gold standard for TB disease diagnosis. Therefore, the sensitivity of QFT-GIT should be evaluated using cases of confirmed TB disease. However, *M*. *tuberculosis* detection is rarely obtained in children. So, evaluating the reliability of IGRA may be difficult, particularly in children [21].

## Conclusion

This study showed that QuantiFERON®-TB Gold In-Tube remains of little contribution to the diagnosis of active TB in children living with HIV in a TB-endemic country. Modifications are needed to improve its performance. QFT-GIT could instead be used in association with the clinical diagnosis of active TB in countries that can afford its use.
